# Effects of Feed Supplementation on Mineral Composition, Mechanical Properties and Structure in Femurs of Iberian Red Deer Hinds (*Cervus elaphus hispanicus*)

**DOI:** 10.1371/journal.pone.0065461

**Published:** 2013-06-04

**Authors:** Cesar A. Olguin, Tomas Landete-Castillejos, Francisco Ceacero, Andrés J. García, Laureano Gallego

**Affiliations:** 1 Animal Science Tech, Applied to Wildlife Management Res.Group, IREC Sec. Albacete, IREC (UCLM-CSIC-JCCM), Campus UCLM, Albacete, Spain; 2 Grupo de Recursos Cinegéticos, Instituto de Desarrollo Regional (IDR), Universidad de Castilla-La Mancha (UCLM), Albacete, Spain; 3 Departamento de Ciencia y Tecnología Agroforestal y Genética, ETSIA, Universidad de Castilla-La Mancha (UCLM), Albacete, Spain; 4 Department of Animal Science and Food Processing in Tropics and Subtropics, Faculty of Tropical AgriSciences – Czech University of Life Sciences, Praha– Suchdol, Czech Republic; University of Western Ontario, Canada

## Abstract

Few studies in wild animals have assessed changes in mineral profile in long bones and their implications for mechanical properties. We examined the effect of two diets differing in mineral content on the composition and mechanical properties of femora from two groups each with 13 free-ranging red deer hinds. Contents of Ca, P, Mg, K, Na, S, Cu, Fe, Mn, Se, Zn, B and Sr, Young’s modulus of elasticity (*E*), bending strength and work of fracture were assessed in the proximal part of the diaphysis (PD) and the mid-diaphysis (MD). Whole body measures were also recorded on the hinds. Compared to animals on control diets, those on supplemented diets increased live weight by 6.5 kg and their kidney fat index (KFI), but not carcass weight, body or organ size, femur size or cortical thickness. Supplemental feeding increased Mn content of bone by 23%, Cu by 9% and Zn by 6%. These differences showed a mean fourfold greater content of these minerals in supplemental diet, whereas femora did not reflect a 5.4 times greater content of major minerals (Na and P) in the diet. Lower content of B and Sr in supplemented diet also reduced femur B by 14% and Sr by 5%. There was a subtle effect of diet only on *E* and none on other mechanical properties. Thus, greater availability of microminerals but not major minerals in the diet is reflected in bone composition even before marked body effects, bone macro-structure or its mechanical properties are affected.

## Introduction

Bone tissue is the major part of the skeleton and one of its main roles is structural function, such as organ protection, locomotion, muscle activity, load-bearing, and serving as a reserve of minerals [1]. Whole bone mechanical properties depend on factors such as cortical thickness, diameter and quality of material [2]. In long bones, the resistance to flexion increases with cortical thickness [1]. The external diameter of long-bones predicts 55% of variation in resistance to flexion [2]. But bone stiffness also depends on intrinsic material properties (*i.e.,* those independent of size and shape) such as porosity, level of mineralization, crystal size, and properties derived from the organic phase of bone [3], [4]. The most widely studied intrinsic mechanical properties include: Young’s modulus of elasticity or stiffness (*E*), bending strength (force required to break a sample of bone), and work to fracture (the work required to produce such break) [4], [5].

Nutrition is a main factor affecting composition of bone. These in turn affect the degree of mineralization and size of bones, both of which influence mechanical performance [5]. In addition to the overall effects of the abundance of food, the mineral profile in diet can influence the mechanical performance of bones. This ranges from the more obvious effect of Ca and P [6], to the more subtle effects of minor minerals (*i.e.* Mg, Mn, Cu, S, Zn [1], [7], [8], [9]). Several studies have assessed the importance of almost all minor minerals by examining their relative deficiency in single-mineral studies ([10], [11] and references therein). However, several recent studies have calculated the relative importance of these minerals by assessing natural variation of both bone mineral composition and mechanical properties in deer antlers. In antlers the mineral profile differed between different parts, reflecting the size and structural quality of the antler and the adequacy of the diet [12], [13], [14], [15], [16]. Furthermore, [13], [14] management affected mineral trends along deer antlers, in turn associated with better mechanical performance of bone material in deer with better nutrition. In one case, a change in content of a minor mineral in the diet produced a disproportionate effect in weight, structure and mechanical properties of antlers [9].

Antlers are bones, but differ from ordinary internal bones in that they grow rapidly [17] and are then cut off from the blood supply, and so effectively die, though their function is still to be tested. They show very little remodelling [18]. Thus, whereas antlers may reflect diet in the recent past, internal bones are more likely to reflect diet in the long term.

The aim of this study was to examine the effects of food supplementation on mineral composition, size, structure, and intrinsic mechanical properties of deer femora. In addition, we also aimed to assess variation in mineral profiles among different parts of the femur. Because (in contrast to antlers) nutrition effects on internal bones may constitute a slow process due to remodelling, we studied animals that had been on the same diet from weaning up to 3 years of age. In order to assess the overall importance of the diet for the growth of the animal, we also examined differences in body size, body condition and weight between groups of hinds.

## Materials and Methods

### Animals and Handling

We studied 26 captive female Iberian red deer (13 with access to supplement food and 13 as control group) from a private game estate in the Ciudad Real province (38°53′N, 4°17′E), Spain. The hinds had been captured as calves at weaning. Ninety percent of Iberian red deer calve over a period of four weeks [19], so that the studied hinds probably differed in age by not more than a few weeks. Animals were kept outdoors in two contiguous fenced enclosures extending over a natural area of 13.5 ha each. All animals were maintained in captivity between 2004 and 2007, when they were hunter harvested (autumn 2007) by gamekeepers at a age of 3.5 years. Because no males lived with them, none of the hinds had been pregnant during the experiment (pregnancy and lactation increase the mobilization and resorption of Ca in the skeleton [20]). No management practice other than daily refilling of feeders was carried out during the experiment.

### Ethical Note

We followed Spanish and European (EU Directive 2010/63/EU for animal experiments) guidelines and laws for the use of animals in research [21]. The experiment was approved by the University Ethical Committee of Universidad de Castilla-La Mancha (no 0610.04).

### Protein and Minerals in Diet: Plant and Supplement Feed Analyses

The first group of hinds was supplementally fed with wholemeal feed (pellet feed) while the second group had access only to the natural vegetation present in the area (natural pasture and shrub-steppe in a Mediterranean forest; protein and mineral compositions for wholemeal feed and natural vegetation are shown in Table 1A). The supplemented group of hinds had permanent access to 1 kg day^−1^ animal^−1^ of pelleted food commonly used in deer private game estates. This is more than the deer usually consumed (*i.e.,* in fact they were fed *ad libitum*). In order to estimate overall intake of protein and minerals in the supplemented group, 2.5 kg dry matter intake (DMI) was assumed based on other studies [22], [23] and the experience in the experimental deer farm at our university. A mean ingestion of 1 kg of supplementary feed per animal per day would account for 40% of total DMI, whereas natural vegetation would account for the other 60%. Thus, the actual daily intake of protein and minerals could be calculated as 0.4*content in supplementary feed +0.6*content in vegetation (Table 1B).

**Table 1:** Mineral and protein content of supplemental feed offered to Iberian red deer hinds at 1 kg individual^−1^ day^−1^, and mean mineral content in main chewed plant species present in the study area.

1A			
Nutrient	Feed	Vegetation	Feed/Vegetation ratio
Crude Protein (%)	22.00	9.51	2.3
Calcium (%)	1.69	0.80	2.1
Phosphorus (%)	0.59	0.11	5.5
Magnesium (%)	0.35	0.24	1.5
Potassium (%)	1.00	0.95	1.1
Sodium (%)	0.37	0.02	18.5
Sulfur (mg/kg)	1295.60	899.20	1.4
Copper (mg/kg)	35.50	5.90	6.0
Iron (mg/kg)	475.50	119.20	3.9
Manganese (mg/kg)	467.10	89.80	5.2
Selenium (mg/kg)	1.72	3.72	0.5
Zinc (mg/kg)	401.10	27.60	14.5
Boron (mg/kg)	11.76	26.18	0.4
Strontium (mg/kg)	29.09	49.64	0.6
Silicon (mg/kg)	4100.00	900.00	4.5
Cobalt (mg/kg)	1.21	0.60	2.0
Molybdenum (mg/kg)	4.15	2.45	1.7
**1B**			
**Nutrient**	**Supplemented Group**	**Control Group**	**Supplemented/Control diet ratio**
Crude Protein (g)	362.7	237.8	1.5
Calcium (g)	28.9	20.0	1.5
Phosphorus (g)	7.6	2.8	2.8
Magnesium (g)	7.1	6.0	1.2
Potassium (g)	24.3	23.8	1.0
Sodium (g)	4.0	0.5	8.0
Sulfur (mg)	2644.4	2248.0	1.2
Copper (mg)	44.4	14.8	3.0
Iron (mg)	654.3	298.0	2.2
Manganese (mg)	601.8	224.5	2.7
Selenium (mg)	7.3	9.3	0.8
Zinc (mg)	442.5	69.0	6.4
Boron (mg)	51.0	65.5	0.8
Strontium (mg)	103.6	124.1	0.8
Silicon (mg)	5450.0	2250.0	2.4
Cobalt (mg)	2.1	1.5	1.4
Molybdenum (mg)	7.8	6.1	1.3

Table 1A shows the composition of the feed and vegetation, as well as their ratio. Table 1B shows overall mean diet composition in both supplemented and control groups, based on 1 kg of supplemental feed +1.5 kg of natural vegetation (*i.e.* 40–60% of diet) in the supplemented group *vs*. a 100% natural vegetation diet in the control group, as well as the intake ratio among groups.

We collected four samples (at the midpoint of each season) of the supplementary feed offered during the experiment and the natural vegetation. Ten plant species common in the study area and previously reported as preferred by Mediterranean red deer [24] were selected and analysed: strawberry tree (*Arbutus unedo*), gum cistus (*Cistus ladanifer*), rockrose (*C. salviifolius*), gum succory (*Chondrilla juncea*), mock privet (*Phillyrea angustifolia*), mastic tree (*Pistacea lentiscus*), purslane (*Portulaca oleracea*), holm oak (*Quercus ilex*), kermes oak (*Q. coccifera*) and purple vetch (*Vicia benghalensis*). Samples (about 200 g) were harvested in 15 different locations in the study area. Leaves and stems were collected since these are the parts preferred by red deer [25]. The samples were dried in an oven (T-Qtech Model 80L, Barcelona, Spain) at 85°C for 48 h, ground and stored as powder. Finally, 10 g from each sample was mixed for mineral and ash analyses. The data obtained in this way were regarded to reflect the mean yearly mineral content in the diet. Samples of supplementary feed were processed and analyzed in the same way. Crude protein was determined with the Kjeldahl method in a digester Pro-Nitro M (JP Selecta, Barcelona, Spain) and evaluated in a 848 Titrino Plus (Metrohm, Switzerland).

### Animal Measurements

The shot animals were transported to a dissection room for data, organ and tissue collection, which took place within 6 h after death.

To assess the effects of diet on body growth and body condition, the following parameters were recorded for each hind: body weight, skin-on carcass weight, kidney weight, kidney fat weight, total body length, femur length, femur cortical thickness (see below for details), chest girth (as described in [26]), and foot length. Kidney fat index (KFI [27]) was calculated as the weight kidney fat divided by kidney weight without fat multiplied by 100. This is an estimator of body condition in deer [28].

### Femur Sample Extraction and Specimen Preparation

**Figure 1:**
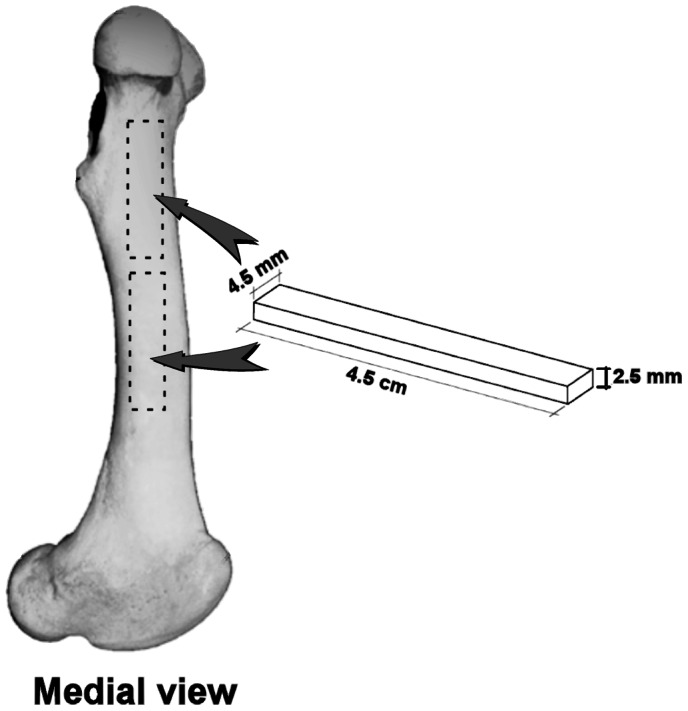
Sections of the femur sampled for chemical analysis (arrows) and mechanical testing (femur bars 45 mm×2.5 mm×4.5 mm indicated in the drawing).

Left and right femora were removed and stored in a freezer until experimental processing. Each femur was then manually cleaned of adhering soft tissue or other material. Femur length was measured with a digital calliper using standard measurement protocols. The complete femur was cut in 3 parts of similar length with a hand-held drill equipped with a saw blade (Dremel Series 3000, Illinois, USA): upper third or proximal part of diaphysis (PD; Figure 1), central part or mid-diaphysis (MD) and lower third (distal diaphysis). For subsequent analyses we used PD and MD because they probably have slightly different functions and so these sections may have both different mechanical properties and mineral composition. Sawing was performed under running tap water to avoid overheating of the bone tissue.

Following sectioning, cortical thickness was measured using a digital calliper. Cortical thickness of PD and MD was measured at four equally-spaced points of the cross sections (Figure 1). At each point five measurements were performed and the mean of these consecutive measurements was recorded. The single value for cortical thickness (MD, PD) used for statistical analysis was the average of the means obtained for the four sites. Thereafter, we extracted sticks from MD and PD which were used first for mechanical testing and then for chemical analysis. The rough-cut sticks were extracted from the internal parts of left and the right femora. The gross sticks were immersed in Hank’s Buffered Salt Solution (HBSS, BioWhittaker) and kept frozen at -20°C until they were processed to produce exact-sized specimens for mechanical testing. The reason we used HBBS is that immersion in non-calcium-buffered saline has been shown to result in a loss of calcium and a 2% reduction in Young’s modulus of elasticity *E* [29], [30]. Although no such changes occur when the samples are kept frozen, we nevertheless used immersion in HBBS instead of water, since they were left to thaw for several hours. Specimens were abraded using a semiautomatic polishing equipment (Struers LaboPol-21, Ballerup, Denmark) until they reached dimensions of 45 (length) × 2.5 (depth) × 4.5 (width mm. Samples were kept moist taking care to produce plane-parallel surfaces with a deviation of smaller than 0.01 mm (ACHA, Digital Caliper, Spain). The exact-sized sticks were again immersed in HBSS and kept in a refrigerator until mechanical testing, replacing the solution every week if necessary. The specimen was marked so the orientation was known. Specimens were always loaded with the periosteal side in tension.

### Mechanical Testing

The mechanical performance (e.g. resistance to fracture) of a complete bone or bone portion depends on two sets of factors [1]: i) architectural ones, mainly depending on cortical thickness and bone diameter in areas such as the bone shaft [2]; ii) mechanical bone properties that have to be tested in specimens of standardized size. Architectural parameters were determined as detailed above. In addition, the following mechanical properties of the material, that is on the intrinsic mechanical properties [1], [3] were determined: stiffness (*E*), bending strength, and work to facture. We tested exact-sized specimens from MD and PD in a three-point bending test machine (Zwick/Roell 0.5 kN, Ulm, Germany). Span length of the supports was 40 mm and speed of the cross head 32 mm min^−1^. Because mechanical properties of the femur, antler and other bones differ greatly depending upon the hydration state [4], great care was taken to keep the specimen fully hydrated right up to the start of the mechanical testing. The machine produced an output chart in the software testXpert II (Zwick GmbH & Co, Ulm, Germany).

*E* was determined from the initial slope of the load-deformation curve between 4 and 10 N, which was usually linear. Bending strength was determined from the maximum load borne. This mechanical property is considered as the relation of bending moment resulting from the mechanical test with the deflection of the sample, multiplied by squared depth and divided by second moment of area [1]. Work to fracture was determined by total work done on the specimen up to the greatest load borne, divided by the ‘central’ cross-sectional area. It is the amount of energy per unit of area required to break bone material, expressed as J m^−2^ [31].

### Chemical Analysis

For chemical analyses, the specimens used in mechanical testing were dried at 60° for 48 h, ground and divided into two subsamples of 0.5 g each (one for assessing ash content and one for mineral content). We used a scale (Gram SR-410M, Barcelona, Spain) with a precision of 0.001 g. Subsamples from natural vegetation and wholemeal were prepared and analysed in the same way.

Samples for ash content were dried in an oven at 105°C for 24 h, weighed, and ashed in a muffle furnace (Hobersal, Model HD-230, Barcelona, Spain) at 550°C for 4 h. Then, samples were cooled and weighed. Ash content was calculated by dividing the ashed weight by the weight of the dry sample, and multiplied by 100.

Samples for mineral content were dissolved with acid solution (12% HCl, 32% HNO_3_ and 56% H_2_O). A second wet digestion was carried out in a microwave oven (Perkin-Elmer Multiwave 3000, Boston, USA) under 345 kPa for 30 min. Then, samples were examined with an atomic absorption spectrophotometer (Optima 5300 DV, Perkin-Elmer ICP-OES, Boston, USA). To assess mineral profile, we analyzed thirteen of the most important minerals: Ca, Mg, P, Na, K, S, B, Cu, Fe, Mn, Se, Sr and Zn. We also included Mo, Co and Si for plant and feed analyses, but there were detection problems of these minerals in femurs for an unknown reason, and so they were discarded for bone, but not for comparisons of diet.

Macro-minerals results are expressed as percentages, whilst micro-minerals are expressed as parts per million (mg/kg).

### Statistical Analysis

Ratios of mineral availability in both groups were calculated in two ways: i) a simple ratio between content of protein and each mineral in supplemental feed *vs*. natural vegetation; and ii) a ratio of total protein and mineral content in the diet of experimental *vs*. control group.

Differences between groups in body weight and body condition (KFI), foot length, body length, thoracic perimeter, femur length, cortical thickness, ash and mineral content, and bone mechanical properties (*E*, bending strength and work to fracture) were examined using one-way ANOVA for those variables which had a single value per hind (*i.e.* body weight); in the case of KFI, using the mean value between both kidneys; and mean of all sampled sites in the femur variables (although on table 2 both right and left mean values are shown). To avoid excessive degrees of freedom with regard to sample size and the complication of too many potential interactions (two levels of repetition: left-right femurs, and centre-upper shaft within each), an ANOVA was performed to assess if left and right femurs differed in composition and mechanical properties. As no variable showed significant differences between left and right femurs, data of both femurs were aggregated into two mean values for each hind: one for MD and one for PD.

**Table 2:** Differences in body properties measured (Panel A) and femur mechanical properties, ash and minerals between two groups of red deer hinds feeding on natural vegetation (control group) or plants plus 1 kg animal^−1^ day^−1^ of food supplement indicated in Table 1 (food supplemented group).

Parameter	Food supplemented group	Control group	P
**A. Body parameters**			
Live weight (kg)	90.1±1.4	83.6±1.6	0.005
Carcass weight (kg)	60.1±1.3	56.4±1.7	0.09
Body length (cm)	161±2	160±4	–
Thoracic perimeter (cm)	111.9±1.3	109.0±1.4	–
Hind foot length (cm)	49.19±0.35	49.81±0.40	–
Weight of left kidney (g)	119.6±4.2	109.9±4.4	–
Weight of right kidney (g)	113.9±4.4	109.9±5.7	–
KFI left kidney (%)	121±13	59±7	0.001
KFI right kidney (%)	145±15	72±12	0.002
Left femur length (cm)	27.54±0.13	27.27±0.26	–
Right femur length (cm)	27.46±0.14	27.27±0.20	–
Left femur cortical thickness (mm)	4.97±0.10	4.95±0.18	–
Right femur cortical thickness (mm)	5.07±0.22	4.73±0.13	–
**B. Femur composition and mechanical properties**			
*E* (GPa)	22.4±0.3	22.0±0.3	–
Bending strength (MPa)	264.2±6.4	271.6±6.0	–
Work to fracture (kJ m^−2^)	9.4±0.4	9.3±0.4	–
Ash (%)	72.3±0.3	72.5±0.2	–
Calcium (%)	27.5±0.1	27.7±0.1	–
Phosphorus (%)	13.09±0.05	13.05±0.05	–
Magnesium (%)	0.447±0.004	0.451±0.003	–
Potassium (%)	0.0282±0.0003	0.0297±0.0003	0.001
Sodium (%)	0.649±0.004	0.659±0.003	–
Sulfur (mg/kg)	555.4±3.2	554.2±3.9	–
Copper (mg/kg)	0.250±0.007	0.231±0.006	0.048
Iron (mg/kg)	1.4±0.8	1.6±0.5	–
Manganese (mg/kg)	0.32±0.01	0.26±0.01	0.001
Selenium (mg/kg)	0.51±0.05	0.42±0.04	–
Zinc (mg/kg)	63.4±1.2	60.0±0.9	0.024
Boron (mg/kg)	2.06±0.05	2.40±0.06	0.001
Strontium (mg/kg)	238.8±5.1	251.1±3.5	0.050

Means ± SE.

General linear mixed models (GLMM) examined effects of supplementation and femur region (MD *vs*. PD) on mechanical properties. Because mechanical properties have been usually explained in terms of Ca content, this was included in the model as a covariable, so that the GLMM could evaluate the effects of supplementation independently of the effect of calcium content in the femur. Individual hinds were entered as the subject, and position in the femur as the repeated measure. All analyses were performed using SPSS 18.0 for Windows (SPSS, Chicago, IL, USA).

## Results

Table 1A shows protein and mineral composition of supplement food and vegetation, and their ratio. The largest ratio between feed and vegetation corresponded to Na and Zn (18.5 and 14.5 times greater in the supplement food, respectively). The greatest ratios after these corresponded to Cu, P, and Mn (6.0, 5.5, and 5.2 times more in feed than vegetation, respectively). Table 1B shows protein and mineral content in the diet in the supplemented group (40% feed +60% vegetation), that in the control group, and the ratio between both. Final ratios in the diet showed: greatest availability in Na and Zn (8.0 and 6.4 times more, respectively), followed by Cu, P and Mn (3.0, 2.8, and 2.7, respectively).

Biometric variables (table 2) showed a significant difference between groups in live weight (supplemented group 90.1±1.4 kg, control group 83.6±1.6 kg; *P* = 0.005) and KFI (supplemented group 131.2±13.7%, control group 65.4±9.9%; *P* = 0.005; table 2 shows left and right values as well as their *P*), but not in carcass weight, thoracic perimeter, body length, femur length, cortical thickness or foot length (table 2). No differences were found between left and right values for PD and MD sections of the femur and thus, left and right values for each region were pooled as a single value for further analyses.

Regarding mean mineral composition and mechanical properties, Table 2 also shows differences between femurs from supplemented and control groups. The greater mineral availability (in ratios feed *vs*. plant content, or between diets) in supplemented group did not affect macromineral femur content (as Na or P). However, greater availability in supplemented diet significantly increased contents of Mn by 23%, Cu by 9% and Zn by 6%. Similarly, the greater content of B and Sr in plants (25% more in the diet based only in plants) may be responsible for the greater content of B and Sr in femurs of hinds under control diet by 14% and 5%, respectively. Finally, despite having the same content of K in both diets, femurs of hinds in the control group had a greater content of K.

No effect of supplementation was found in ANOVAs testing mechanical properties. However, detailed GLMM with repeated measures on mechanical properties including Ca as a covariable showed subtle effects that the ANOVA could not show: 1) the supplemented hinds had bone material with 27.2 GPa additional stiffness (*E*); 2) there was a Ca effect in the control but not in the experimental group, so that in the control group femurs increased stiffness when the amount of Ca increased in this bone (model intercept = 28.9±9.3; coefficient for control group = −27.2±12.3, *P*<0.05; interaction coefficient only for control group Ca = 0.73±0.29, *P*<0.05). No other effect of supplementation was found in mechanical properties. No effect of femur region (MD *vs*. PD) was found either.

## Discussion

The results showed that a greater availability of several major and minor minerals in an enriched diet was reflected in internal bone composition only in the minor minerals with greatest availability, but not in major minerals such as Na or P. Moreover, this effect occurs at a moderate level of supplementation producing only slight changes in live weight and body condition at macrostructural level (probably produced by the greater availability of protein and energy in the supplement), but not changes in body growth or bone structure at largest scale. Thus, long term availability of minerals in the diet seems to be reflected in bone composition only in micro but not macrominerals.

A first step to understand how important the level of supplementation was is to assess its effects on general body weight and size, as well as the effects on internal organs and bone size. Despite being under the nutrition scheme for 3 years, just after weaning, hinds only showed a difference of 6.5 kg between groups and a two-fold difference in KFI, but no difference in body or femur size. The 7.2% difference in weight as a result of food supplement is similar to figures reported by Peek *et al*. [32], who found a mean weight increase of 9.8% in hinds feeding open range with supplements compared with hinds without supplements. In the present study, the differences between groups in KFI showed that supplementation improved body condition significantly, although not markedly to the observer’s eye (in contrast to other published studies [33], [34], [35]). It should be noted that the differences cannot be solely attributable to mineral composition, as most likely they are derived from the greater amount of protein in the supplemented diet, their greater digestibility, as well as fat content, energy, and other nutrients whose measure was beyond the scope of this article.

The weight difference in our study may seem a remarkable difference, but in fact it is not a marked effect for nutrition in deer. Our own experiments showed a 10 kg difference in a group under a 60% restriction in diet compared to a control group after just 10 weeks during lactation [36]. Moreover, unpublished records in our experimental farm under *ad libitum* diets show more than 50 kg difference between adult hinds of the same nutrition level. Indeed, the standard error of the KFI shows an 87% variation with respect to the mean in body condition in the supplemented group, and only 15% in the control group, which also suggest a large variation within the supplemented group. Unfortunately, the experimental set-up with hinds in a nearly free-living situation did not allow us to estimate individual intake of plants (and species composition) or feed. Thus, we could not study further the causes for such large variation of KFI and other variables in the food supplement group.It is even more surprising that no effect was found for growth. Previous studies by our group have shown that a 3-month advance of calf births led to an 11 kg difference between groups one year later despite there being no difference in lactation and having food *ad libitum* [37]. This is not only a weight difference: differences during lactation at low or standard milk production resulted in significant weaning differences of 7% in thoracic perimeter, 5% in cranial length, and 3% in height at shoulders [38].

Thus, it is remarkable that supplementation affected the mineral composition and to some extent mechanical properties of internal bones, even in a setting that produced slight effects on live weight and body condition, and no effects on growth, cortical femur thickness, its length or ash content. We cannot rule out that further studies analyzing the micro-structure of femurs using micro-CT and back scattered electron microscopy to assess the distribution of mineral at microscopical level, or other fine-detail techniques used in antlers [18], [39] could reveal differences. These studies should be very interesting, but they were beyond the scope of our aims. Such studies could also benefit greatly by assessing a wide arrange of nutrients in the diet apart from crude protein (fat content, energy, fibre, etc.).

The results show an increase in mineral content in femur of 23% in Mn, 9% in Cu, and 6% in Zn associated with supplementation. The supplement contained, by order of magnitude, 18.5 times more Na than in natural vegetation, 14.5 for Zn, 6 for Cu, 5.36 for P, and 5.2 for Mn. Considering a 2.5 kg feed intake per animal and day being 40% of the diet, the supplemented group had 8 times more Na, 6.4 times more Zn, 3 times more Cu, 2.8 times more P and 2.7 more Mn. In both ratios the differences found in femur reflected those of the diet except for Na and P. One of the differences between Na or P and the other minerals mentioned is that Na and P are macro-minerals. Perhaps microminerals are reflected more or less directly in the bone whilst this is not true for macrominerals. This may point to the role of bone as a store of microminerals. The literature shows contradictory evidence: some early studies have shown that internal bones do reflect the level of Ca, P, and Mg [40], [41], [42]. However, it has been found in deer that Zn and Mn increased in several tissues in direct proportion to the content in dietary feed [23]. In contrast, Na and P in the diet may not be directly reflected in bone as a result of threshold effects. In fact P and Na, but also Ca and Mg showed a similar concentration in hind femurs as published values for human cortical bone [38]. The greatest availability in supplemented diet after Zn, Cu, and Mn are Ca and Fe. Of these, only Fe is a micromineral and it is not clear why their content in the diet is not reflected, but after these, the following are three minerals showing 25% more availability in the control diet: B, Sr, and Se. Of these, the first two were also reflected in a greater content of B and Sr in the femurs of hinds under the control diet. That is to say, except for Fe and Se, differences in femur micromineral composition reflect their availability in the diet (greater content in femur if the diet has a greater content). Contrary to this, of the major minerals femurs only differed in K, and this despite its content in the diet being the same.

Why did femurs not reflect mineral availability of Na in the diet? Research in other cervids has shown that moose (*Alces alces*) select plants for Na content to meet a Na threshold, and thereafter, the diet is selected for energy content [43]. A similar effect of seeking for Na when it is deficient has been frequently found in red deer [44]. In contrast to our findings here, Na has been found to reflect diet composition in antlers [14], [15]. However, in this case, it reflected a deficiency, which suggests that bone Na may reflect diet only in a deficiency situation and only up to the point in which needs have been fulfilled. This would not be surprising, as animals are able to modulate mineral absorption according to their needs, reducing absorption when needs have been met [45]. This, in turn, would also support the hypothesis that internal bones reflect diet only in microminerals as a store to be subsequently liberated by remodeling when they are needed. Recently, a hypothesis has been put forward by our group to explain remodeling as a mechanism to keep flow of microminerals from skeleton to other organs where they are needed [46].

However, the percentage of increase in Mn, Cu and Zn between femurs of supplemented and control groups do not match the ranking in the ratio of these minerals in the diet (in other words, in bones the ratio is similar for Mn and Cu and far less for Zn, whereas it is far greater for Zn in food and similar in the other two). Up to some extent this is not surprising for two reasons: the ratio between content in supplemental diets is based on the assumption of equal intake of all plant species as we did not have information on the percentage of each species in the real hind diet. The second reason is that absorption or bioavailability of a mineral may depend on the interaction between physiological importance of the mineral and its availability in the diet. Thus, animals may store all Mn or Cu they can at relatively low contents, whereas the more concentrated Zn in our setting may have a much lower priority for storing. At least for the case of Cu, its mere addition to an otherwise balanced diet may increase growth in pigs [47], and in fact, it is commonly deficient in ruminants ([48]; Ludek Bartos, John Fletcher and other deer scientists personal communications).

Once discarding Si which unfortunately we could not measure well in bones, the remaining ranking in mineral availability in feed *vs.* plants, corresponds to Fe, Ca, B, Se, Mg and Sr. Of these, Ca and Mg are again two major minerals whose greater availability in the diet are not reflected in femurs. Of the remaining, which are minor minerals, Fe and Se are not reflected in their content in bones, but B and Sr are. B may be absorbed with a greater priority to counteract the lack of other minerals in the bone, as it has been shown to improve bone mechanical properties [49], [50]. Some authors reported that B and Sr were associated with increased ash content in bones [23], [51], which may explain why hinds in the control group incorporated more of these minerals in their femurs, thus reflecting the diet in contrast to Se, and why we did not find differences in ash content between groups. Se is mainly not stored in bones but in muscles, leading to white-muscle disease if it is deficient [11]. This may be the reason why bone does not reflect Se content of the diet. We do not have an explanation on the lack of effect of Fe in bone.

In contrast, it seems easier to explain why control group had femurs with 5% more K despite having nearly the same availability of K in the diet. The greater K content in femurs of hinds feeding only on plants may indicate that these are under a greater nutritional stress. Studies on antler show that K indicates physiological stress in this kind of bone, as its content is greater in distal parts of the antler which are grown when body stores of minerals are near depletion [13], [15]. Similarly, another influence of diet upon antler composition (in this case milk production effects on mineral composition of first deer antler), also pointed to a relation between K and nutrition stress: the lower the milk production by the mothers, the greater the K content in first antlers, whereas the opposite was true for antler ash, Ca and P content [52].

With regard to mechanical properties, we found an effect of supplementation only in stiffness (*E*), and this could only be assessed after removing the effect of Ca. How can stiffness, usually related to Ca, be increased in the experimental group once the effect of this mineral and femur region is removed? In chemical terms, in the appatite lattice that forms the mineral phase of bone Ca can substituted for Mg or Sr, but also by other minerals with similar valency. This may have happened in our case, or the effect may be caused by other nano-scale factors. To assess further this question we should test also ratios such as Ca/Mg, Ca/Sr, and other as we did in a previous study [13], but this is a matter for a paper more focused on the effects of chemistry on mechanics at nano-scale, rather than the main effects of diet in bone composition and mechanical properties. The limited effect found of supplementation on bone mechanical properties compared to effects found in antlers [13] may indicate that internal bones, and particularly long bones which sustain the body weight, have more conserved mechanical properties than those antlers. The reason may be that breaking a leg, for example, has more serious consequences for survival than breaking an antler: the former usually ends in death of the animal. However, and as pointed above, we cannot rule out that more subtle effects could be found if other intrinsic mechanical properties are found in test regarding stretching, shear, compression, or hardness tested by micro or nano-indentation. The diet may have affected the distribution of minerals at the microscopical level, which in turn may influence mechanical properties, or may influence porosity [18], [39], which is also directly related to some mechanical properties [39]. Thus, further analysis based on these techniques may be very interesting to complete the present study.

If the present results were found in other situations, they may be potentially useful both for deer farming and ecological studies. For example, a chemical analysis of all plants present in an ecosystem may not show gross differences in mineral availability. However if a study finds that two populations of deer feeding on plants of their respective habitats differed, for example, in femur content of Mn, B, Zn or other minor minerals, this would mean that the plants actually ingested by deer do actually differ in mineral availability between populations. Thus, examining the bone composition of a population of deer and comparing it with that of deer under a balanced diet may be a tool to suggest that mineral supplementation should be considered to correct mineral deficiencies. Because threatened deer or indeed other ruminants cannot be killed or caught for detailed physiological studies, this tool could also be used collecting bones from individuals found dead to compare with zoo animals to detect mineral deficiencies in natural populations that may be the cause of the declining of a wild population.

### Conclusions

Supplemented food involving greater mineral availability can influence internal bone composition, mostly regarding the microminerals more available in supplement food. This effect occurs even in healthy animals and when supplemented deer only achieved moderate difference in weight or body condition with the control group, but no difference in body growth, femur size, cortical thickness or mechanical properties.
